# Graphene-assisted biosensing based on terahertz nanoslot antennas

**DOI:** 10.1038/s41598-019-46095-x

**Published:** 2019-07-05

**Authors:** Geunchang Choi, Sung Ju Hong, Young-Mi Bahk

**Affiliations:** 1 Department of Energy Science, Sungkyunkwan University, Suwon, 16419 Republic of Korea; 2 Department of Physics, Incheon National University, Incheon, 22012 Republic of Korea

**Keywords:** Imaging and sensing, Optical properties and devices, Metamaterials

## Abstract

We report on improvement of sensitivity for molecular detection utilizing terahertz time domain spectroscopy. Based on confining and enhancing electromagnetic field with metallic nanoslot antennas, we additionally employ monolayer graphene sheet whose edge and hydrophobic surface nature lead to increase detecting performance. Terahertz transmittance in monolayer graphene/metallic nanoslot structure exhibits more unambiguous change after lactose molecules are attached, compared to that in metallic nanoslot structure without monolayer graphene. We attribute the prominent change to that more lactose molecules are guided inside/near the metal gap region due to edge and hydrophobic surface nature of monolayer graphene. This monolayer graphene/metallic nanoslot structure can be expanded in other organic or bio-molecular detection.

## Introduction

Many molecules which have intermolecular interaction exhibit resonant absorption in terahertz frequency regime. Because of millimeter wavelength of terahertz electromagnetic field, large-size pellet is required to study the light-matter interaction. Recently, there has been conducted to sensitive molecular sensing in terahertz frequency range such as explosive molecule, bio-molecule, and microorganism, utilizing large field enhancement and confinement of metallic nanostructures^1–4^. The usage of metallic nanostructure is a fascinating way to improve the interaction where the molecules with relevant strength of the light-matter interaction can be probed with low quantity, since the millimeter wave entirely funnels through the metal gap where the molecules exist^1,5,6^. Furthermore, designing metallic structure to match with resonant absorption of desired materials provides a platform for ultrasensitive detection of specific molecule^1,3^.

In this detecting process, one remaining issue to increase sensitivity is putting target molecules inside/near the metal gaps. Since the terahertz absorption/sensing is proportional to the number of molecule inside/near the metal gaps when the field enhancement of metallic gaps is identical by a fixed gap width, it is important to guide the molecules inside/near the gap. On the one hand, monolayer graphene (MLG) can promote molecular adsorption at its defective site which is relevant for functionalization with various materials from gases to bio-molecules^7–17^. One of the representative defective sites is MLG edge that acts as an effective adsorption agency. In addition to the active site for molecular adsorption, hydrophobic surface nature of MLG may provide higher probability to host the target molecules inside metal gap region because the hydrophobic surface prevents the solution from spreading away. Furthermore, the fact that MLG is compatible with metallic nanoslot antenna without any loss in terms of electric-field enhancement is favored to combine metallic nanoslot antenna and MLG to investigate light (terahertz wave)-matter (ultralow amount of molecules) interaction and develop related sensing devices.

In this work, we combine the two concepts i.e. electric-field enhancement and MLG to increase sensitivity of molecular detection. The MLG/metal gap structure results in synergy in the aspect of electric-field enhancement and increasing the number of molecule near the gap where electromagnetic waves are strongly enhanced and confined. We fabricate nanoslot antenna array conformed by MLG. The MLG/metal gap structure exhibits improved sensitivity for lactose molecules compared to nanoslot antenna without MLG. We attribute the result to the fact that the total number of lactose molecules increases near the gap due to edge structure and hydrophobic surface nature of MLG. Consequently, we elevate sensitivity (or detecting performance) combining the enhanced electric-field and effective attachment of lactose molecules near the enhanced field region, decreasing the detectable molecular density in solution.

## Results and Discussion

Terahertz time-domain spectroscopy is a useful tool for detecting bio-molecules which have resonant absorption in the terahertz frequency regime. We perform the terahertz time-domain spectroscopy using an oscillator-based femtosecond Ti:sapphire laser system with 800 nm center wavelength, 80 MHz repetition rate, and 130 fs pulse width. The laser is divided into terahertz generation and probe beam. Single-cycle of terahertz pulse generated by biased photoconductive antenna with generation beam is guided and focused on samples by off-axis parabolic mirrors. Transmitted terahertz waves are detected through electro-optic sampling using 〈110〉-oriented 1 mm-thick ZnTe crystal with probe beam. We apply the transmission type of terahertz time-domain spectroscopy to detect lactose molecules as a prototypical bio-molecule which has intermolecular absorption in the terahertz frequency.

**Figure 1:**
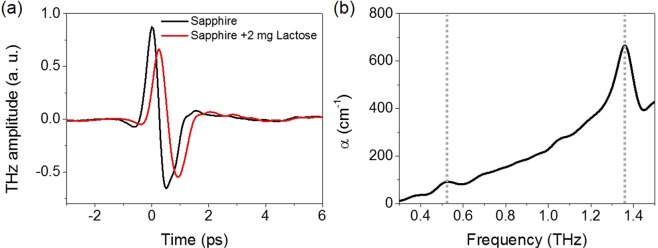
(**a**) Terahertz time traces for bare sapphire (black) and 2 mg of lactose over an area of 3 mm × 3 mm on the bare sapphire (red). (**b**) Absorption coefficient *α* of lactose molecules extracted from the terahertz transmission data, as a function of frequency. The lactose has weak and strong absorption peaks at 0.53 THz and 1.35 THz, respectively (dash lines).

Figure 1(a) shows time traces of terahertz electric-field transmitted through bare sapphire (black) and droplet of lactose solution (total amount of 2 mg) over an area of 3 mm × 3 mm on bare sapphire substrate (red). As shown in the frequency domain (Fig. 1(b)), the lactose molecules have weak and strong resonant absorptions at 0.53 THz and 1.35 THz, respectively, which are consistent with previous works^1,18^. Here, since our purpose is the sensitive molecular detection, we focus on the frequency of 0.53 THz that has smaller absorption coefficient.

**Figure 2:**
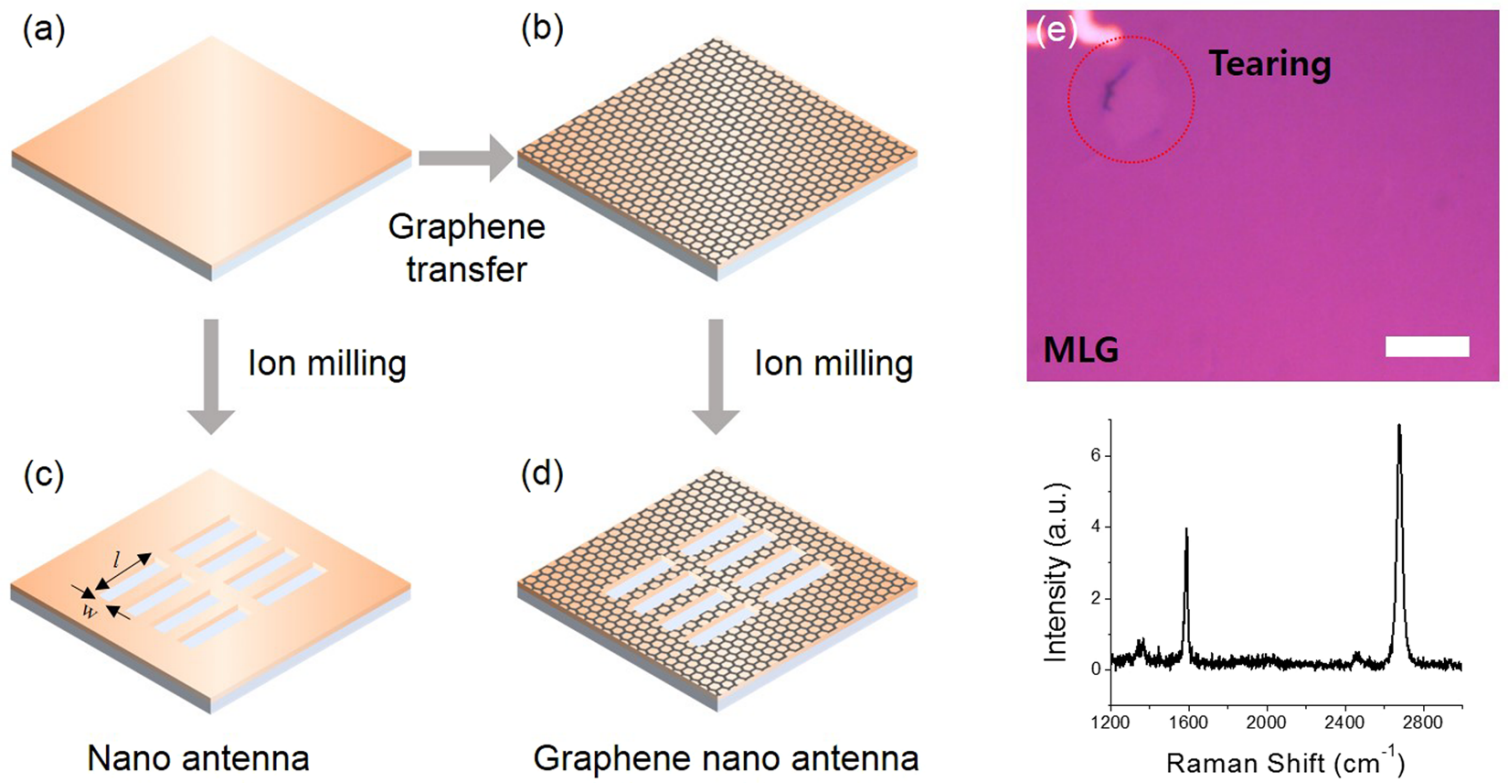
(**a**–**d**) Fabrication process of graphene-based terahertz nanoslot antenna. First, 100 nm-thick Cu films without (**a**) and with (**b**) MLG are prepared. Nanoslot antennas are patterned by focused ion beam on the Cu film (**c**) and the MLG-covered Cu film (**d**). Length (*l*) and width (*w*) of slot antenna are 150 μm and 130 nm, respectively. (**e**) Optical microscope image of MLG on SiO_2_/Si substrate (top) and Raman spectrum of MLG sheet using 532 nm wavelength excitation (bottom). The scale bar is 10 μm.

To obtain ultrasensitive molecular detection using MLG, we constructed two different samples which are arrays of nanoslot antenna with and without MLG. First of all, two 100 nm-thick copper (Cu) films on sapphire substrates are prepared. After then, one of them remains bare Cu film (Fig. 2(a)) and the other is covered by MLG grown by chemical vapor deposition (CVD) method (Fig. 2(b)). We have synthesized large-area MLG on Cu foil (Alfa aesar 13382) at 1070 °C under atmospheric pressure^19^. Subsequently, we transferred MLG on to desired substrate by bubbling transfer method with a 0.1 M NaOH solution^20^. A representative optical image of MLG on SiO_2_ (285 nm)/Si substrate is shown in top of Fig. 2(e). Bottom (lower panel) of Fig. 2(e) exhibits Raman spectrum of MLG sheet using an excitation laser with 532 nm wavelength. Typical G (~1587 cm^−1^) and 2D (~2679 cm^−1^) peaks are observed, where the intensity ratio (I_2D_/I_G_ > 1) and single Lorentzian shape of 2D peak confirm the number of layers^21^. We also observed D (~1350 cm^−1^) peak of which low intensity indicates high quality of MLG sheet. Subsequently, arrays of rectangular hole with a length (*l*) of 150 μm and a width (*w*) of 130 nm were fabricated by focused ion beam (FIB). The resonance frequency of slot antennas is matched to the weak absorption frequency of lactose molecules (0.53 THz). Figure 2(c,d) indicate the final structures of metallic nanoslot antennas without and with MLG, respectively.

**Figure 3:**
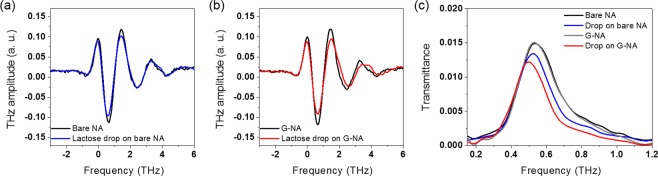
(**a**) Terahertz time traces of bare nanoslot antennas (bare NA) with (blue) and without (black) lactose droplet. (**b**) Terahertz time traces of MLG-covered nanoslot antennas (G-NA) with (red) and without (black) lactose droplet. (**c**) Transmission spectra for bare and MLG-covered nanoslot antennas, with and without lactose droplet.

Figure 3(a) represents terahertz time traces transmitted through nanoslot antennas before (black) and after (blue) dropping small amount of lactose (0.04 μg) solution, showing the distinguishable transmission reduction due to intermolecular absorption of lactose molecules. The corresponding transmission spectra represent reduction of the maximum value of transmittance from 0.0149 to 0.0134 (−Δ*T*/*T*_0,max_ ~ 0.10) (Fig. 3(c)). This has been explained with the absorption enhancement caused by the strong electric-field enhancement in metal nanogap structures^1,22–26^. In addition to the intensity reduction, the resonant peak position moves toward lower frequency (from 0.538 THz to 0.525 THz (−Δ*f*/*f*_0,res_ ~ 0.024)) due to the increasing of the refractive index inside/near the gap by the molecules^3,27,28^. However, it is still required that the sensitivity of terahertz molecular detection is improved for ultra-small number of molecules.

In case of MLG-covered nanoslot antennas, the reduction of terahertz signal is more prominently observed as shown in Fig. 3(b). It is worth to note that the transmission spectrum of MLG-covered nanoslot antennas before molecular dropping (gray line in Fig. 3(c)) has the almost same resonance strength and frequency with that of bare nanoslot antennas (black line in Fig. 3(c)). This implies that the MLG-covered metal structure provides same electric-field enhancement (~630 at the resonance) which determines the detection sensitivity. Note that the field enhancement is extracted by square root of transmittance divided by gap-coverage ratio. Despite the same field enhancement, the MLG-covered nanoslot antenna provides around two times larger transmission reduction (−Δ*T*/*T*_0,max_ ~ 0.18) and red-shift (−Δ*f*/*f*_0,res_ ~ 0.059). This indicates that MLG covering the metal surface of nanoslot antennas boosts the sensitivity of molecular detection with increasing the effective refractive index inside/near the gap caused by more contribution of molecules. From the data, it can be estimated that total number of molecules inside the gap for MLG-covered nanoslot antennas is around two times larger than that for bare nanoslot antennas. We attribute this improved sensitivity to more contribution of molecules caused by MLG which has properties (defective site at edge and hydrophobicity), while higher sensitivity of molecular detection had been achieved by stronger field enhancement and desirable metal structures in other previous works^1–3^.

It is well known that the edge is more active than basal of MLG sheet. In the chemical point of view, the reactivity is determined by electron distribution depending on atomic structure. Compared to intact aromatic structure in basal of MLG, the edge of MLG consists of radicals which possess active electrons. As a result, edge-preferred chemical reactivity can occur^15^, which agrees with the fact that organic molecules favor to attach with defective site of MLG^16^. Therefore, the portion of attached lactose molecules in MLG-covered nanoslot antennas is assisted by the MLG edge which is active site for organic-molecular adsorption with the increase of sensitivity.

**Figure 4:**
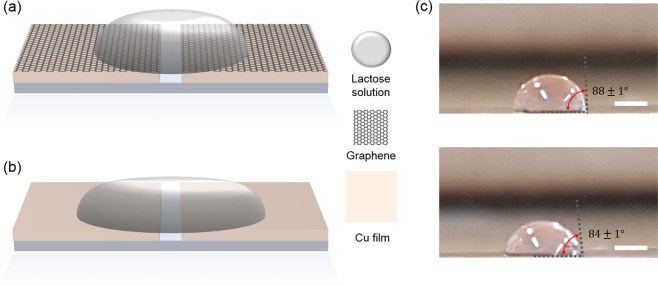
Schematics of lactose solution droplet on different surface nature, (**a**) hydrophobic (Cu with MLG) and (**b**) hydrophilic (Cu without MLG). Total amount (volume) of lactose solution is same for both cases. Since the droplet on hydrophobic (hydrophilic) surface spreads narrower (wider), the height of the droplet at metallic slot antenna is higher (lower). (**c**) The droplet images of lactose solution on Cu/MLG film (top) and Cu film (bottom). The scale bar is 1 mm.

Other effect for increasing detection molecule can be attributed to the hydrophobic nature of MLG. MLG in ambient has been expected to have a surface with hydrophobic nature even though the MLG shows the transition effect from hydrophobicity to hydrophilicity under specific condition such as gate voltage and laser irradiation^29–33^. Schematic diagrams in Fig. 4(a,b) show that the shape effect of lactose solution droplet on hydrophobic (Cu with MLG) and hydrophilic (Cu without MLG) substrates with same total amount of lactose solution, respectively. The surface nature affects contact angle which determines height and area of droplet. Compared to the hydrophilic case, hydrophobic case has larger contact angle of droplet that increases height and decreases area of droplet on the surface. With same lactose concentration and total amount of solution, the height of droplet can be scaled as amount of lactose molecule per unit area. Therefore, more lactose molecules can be present inside/near nanoslot antenna with MLG due to the hydrophobic surface nature. The hydrophobic surface also has a benefit to reduce inevitable coffee-ring effect which suppresses sensitivity due to undesired molecular deposition along the perimeter of droplet. Smaller area of droplet can reduce adsorption-loss originated from the coffee-ring effect out of nanoslot antenna.

Even though quantitative analysis for the adsorption process in microscopic point of view is additionally required, the origins addressed above seem to be in agreement with our observation. Since the solution drop-casting was conducted by micro-pipet in ambient condition, we measured the contact angle of the droplet under the same conditions as drop-casting. The MLG has hydrophobic surface nature with about 88 degree of contact angle, while Cu has hydrophilic properties that decrease the contact angle of droplet about 84 degree (Fig. 4(c)). Larger contact angle represents smaller area and higher solution droplet, increasing the possibility of molecular adsorption near and inside the slot antenna. This is consistent with our terahertz transmission results in Fig. 3 that molecular absorption increases in nanoslot antenna with MLG. We note that our sensing approach can be improved by additional chemical treatment such as fluorine which has larger contact angle^29^.

## Conclusion

In summary, we have investigated lactose molecular sensing using metal nanogap-based terahertz time-domain spectroscopy. We have endeavored to improve the sensitivity with increasing light-matter interaction. The strategies have been achieved by metallic nanoslot antenna array covered by MLG, where we expect two distinct advantages such as (i) enhancement and confinement of electromagnetic waves and (ii) more adsorption of lactose molecules to the nanoslot antenna. Employing MLG conformal to the metallic nanoslot antenna not only maintains the electric-field enhancement but also increases amount of lactose molecules inside/near the gap. The latter can be achieved by adsorption favor of organic molecules on MLG edge and positioning larger number of molecules using hydrophobic surface nature of MLG sheet. We expect that our hybrid graphene-metal nanogap structure opens up increasing sensing probability and can be applied for highly sensitive sensor based on bio- and chemical-solution.
