# Author Correction: Graphene-assisted biosensing based on terahertz nanoslot antennas

**DOI:** 10.1038/s41598-019-55815-2

**Published:** 2019-12-11

**Authors:** Geunchang Choi, Sung Ju Hong, Young-Mi Bahk

**Affiliations:** 1 Department of Energy Science, Sungkyunkwan University, Suwon, 16419 Republic of Korea; 2 Department of Physics, Incheon National University, Incheon, 22012 Republic of Korea

Correction to: *Scientific Report* 10.1038/s41598-019-46095-x, published online 05 July 2019

In Figure 3(a) and (b), the x-axes are labelled wrong, where ‘Frequency (THz)’ should read ‘Time (ps)’. The correct Figure 3 appears below as Figure 1.

**Figure 1:**
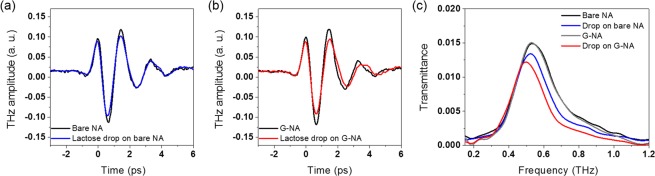
.

